# Crystal Structure and Molecular Imaging of the Na_v_ Channel β3 Subunit Indicates a Trimeric Assembly*

**DOI:** 10.1074/jbc.M113.527994

**Published:** 2014-02-24

**Authors:** Sivakumar Namadurai, Dilshan Balasuriya, Rajit Rajappa, Martin Wiemhöfer, Katherine Stott, Jurgen Klingauf, J. Michael Edwardson, Dimitri Y. Chirgadze, Antony P. Jackson

**Affiliations:** From the ‡ Department of Biochemistry, University of Cambridge, Tennis Court Road, Cambridge CB2 1QW, United Kingdom,; the § Department of Pharmacology, University of Cambridge, Tennis Court Road, Cambridge CB2 1PD, United Kingdom, and; the ¶ Institute of Medical Physics and Biophysics, University of Münster, Robert-Koch Strasse, 31 48149 Münster, Germany

**Keywords:** Analytical Ultracentrifugation, Atomic Force Microscopy, In Vivo Imaging, Sodium Channels, X-ray Crystallography, Photoactivated Localization Microscopy, Protein Trimerization

## Abstract

The vertebrate sodium (Na_v_) channel is composed of an ion-conducting α subunit and associated β subunits. Here, we report the crystal structure of the human β3 subunit immunoglobulin (Ig) domain, a functionally important component of Na_v_ channels in neurons and cardiomyocytes. Surprisingly, we found that the β3 subunit Ig domain assembles as a trimer in the crystal asymmetric unit. Analytical ultracentrifugation confirmed the presence of Ig domain monomers, dimers, and trimers in free solution, and atomic force microscopy imaging also detected full-length β3 subunit monomers, dimers, and trimers. Mutation of a cysteine residue critical for maintaining the trimer interface destabilized both dimers and trimers. Using fluorescence photoactivated localization microscopy, we detected full-length β3 subunit trimers on the plasma membrane of transfected HEK293 cells. We further show that β3 subunits can bind to more than one site on the Na_v_ 1.5 α subunit and induce the formation of α subunit oligomers, including trimers. Our results suggest a new and unexpected role for the β3 subunits in Na_v_ channel cross-linking and provide new structural insights into some pathological Na_v_ channel mutations.

## Teaser

**Background:** The vertebrate sodium channel β3 subunit regulates channel behavior.

**Results:** The immunoglobulin domain of the human β3 subunit crystallizes as a trimer, and the full-length protein assembles as a trimer *in vivo*.

**Conclusion:** Our results reveal an unexpected organization of the β3 subunit.

**Significance:** A new structural insight into the sodium channel is presented.

## Introduction

Sodium (Na_v_) channels initiate the action potential in electrically excitable cells. They are major pharmacological targets and are implicated in pathologies such as cardiac conduction diseases, epilepsy, and chronic pain syndromes (1–3). The vertebrate Na_v_ channel is composed of an ∼250-kDa α subunit, which contains the ion-selective pore, together with ∼40-kDa β subunits that modulate the voltage sensitivity, gating kinetics, and trafficking of the channel. In humans, there are 10 α subunit and four β subunit genes, which are expressed in distinct but overlapping tissue-specific patterns. The Na_v_ β subunits (β1 to β4) are related to the L1 family of cell adhesion molecules and are thought to possess a single extracellular V-type Ig domain, connected via a stalk to an α-helical transmembrane domain and an intracellular carboxyl-terminal region (4–6). The Ig domain contacts the Na_v_ α subunit and modulates its gating properties (4). The Na_v_ β subunits also bind to a variety of cell adhesion molecules such as NF-155, contactin, tenascins, and NrCAMs. These contacts are critical for neurite growth, and some may occur independently of any interaction with the Na_v_ α subunits (6–9). The β subunits thus have multiple roles in normal development, and their potential as therapeutic targets is currently under investigation (5).

We are studying the role played by the β3 subunit in Na_v_ channel structure and function (10–14). In mice, deletion of the β3 subunit gene (*Scn3b*) is associated with cardiac arrhythmias (14, 15). Human patients with *SCN3B* mutations show similar inherited cardiac conduction abnormalities (4). To provide a better understanding of the β3 subunit, we have investigated its structure using the combined approaches of x-ray crystallography and single molecule resolution imaging. We show that the β3 subunits can trimerize via their Ig domains and induce the formation of Na_v_ channel α subunit oligomers, including trimers. Our results have important and general functional implications for the study of Na_v_ channels and their pathologies and provide a new interpretation of previous electrophysiological data that involve Na_v_ β3 subunits.

## EXPERIMENTAL PROCEDURES

### 

#### 

##### Cloning and Expression of β3 Ig Domain

A cDNA clone encoding the β3 Ig domain covering the amino-terminal endoplasmic reticulum (ER)^9^ targeting signal and the carboxyl-terminal hexa-His tag (127 amino acids in total, theoretical molecular mass of 14.8 kDa) was cloned into the mammalian expression vector pTT3 as described previously (13). HEK293F cells were transiently transfected following the manufacturer's instructions. The cells (500 ml) were pelleted at 120 × *g* for 3 min. Medium containing the secreted β3 Ig domain was buffered with 25 mm Tris-HCl, pH 7.7, 0.4 m NaCl and filtered through a 0.45-μm membrane. The filtered medium was applied to a nickel-Sepharose column (HisTrap HP column (Amersham Biosciences, 17-5247-01) in equilibration buffer (25 mm Tris-HCl, 0.4 m NaCl, pH 7.7) and washed extensively with equilibration buffer. The β3 Ig domain was eluted with equilibration buffer containing increasing steps of 10, 20, 40, 50, and 100 mm imidazole. Samples eluted at the 40, 50, and 100 mm steps were pooled and separated by gel filtration using Superdex 75 (flow rate 0.5 ml/min). Protein was checked for purity using 12% SDS-PAGE and concentrated by ultrafiltration to 5 mg/ml.

##### Crystallization

The β3 Ig domain was deglycosylated using peptide:*N*-glycosidase F digestion as described previously (13). Sitting-drop vapor diffusion crystallization trials (total volume of 400-nl drops, 1:1 ratio of protein/precipitant) were set up in MRC 2-drop 96-well crystallization plates using a Phoenix crystallization robot (Art Robbins Instruments, Inc.). A number of crystallization trials with various crystallization screening kits were run; they were all incubated at 19 °C and monitored in a Rock Imager 500 (Formulatrix, Inc.) automated imaging system. Only one condition produced crystals, condition number 16 of the Wizard Classic 2 protein crystallization screen (Emerald BioSystems Inc.) containing 1 m sodium citrate tribasic, 0.1 m CHES/sodium hydroxide, pH 9.5. Crystals having the shape of rods with hexagonal cross-sections started to appear on day 3 and were fully grown after about 3 weeks to a size of ∼0.40 × 0.40 × 0.17 mm^3^. The crystals were harvested directly from the mother liquor and flash-frozen in liquid nitrogen without the use of any cryoprotectant compounds.

##### Data Collection and Processing

The x-ray data collection experiments were performed at a temperature of 100 K at the Diamond Light Source Synchrotron Science Facility (Oxford, UK), beamline I04. The crystals diffracted to a maximum resolution of 2.5 Å (the resolution cutoff level was set to the resolution shell where the average *I*/σ of the reflections is still greater than 2). A total of 100° of data were collected at a 1.0° oscillation angle. The crystals belonged to the P3_1_21 space group and contained three molecules of the β3 Ig domain in the asymmetric unit. This results in ∼50% crystal solvent content (Matthews coefficient (16) of 2.47). All diffraction data were indexed, scaled, and merged using XDS software suite (17). The crystallographic data collection statistics are shown in Table 1.

##### Structure Solution and Refinement

The structure of the β3 Ig domain was solved by the molecular replacement (MR) method. The positions of the three β3 Ig domain molecules within the asymmetric part of the unit cell were identified. All MR calculations were performed in PHASER, part of the PHENIX crystallographic software suite (18). For the identification of the MR search probe, the homolog search was performed using the FUGUE sequence-structure homology recognition server (19). The top three structurally homologous candidates (Protein Data Bank codes 2X1X (20), 1I8L (21), and 1F97 (22)) were clearly separated from the rest of the homologous structures, based on their Z-scores. The homologous regions of the structures corresponding to the β3 Ig domain sequence were cut out (residues 226–326 in 2X1X, residues 1–104 in 1I8L, and residues 29–129 in 1F97) and superposed onto the highest ranking homolog, *i.e.* 2X1X. The superposition resulted in a root mean square deviation of 1.8 Å between equivalent Cα atoms of fragments from 2X1X and 1I8L and 1.6 Å between 2X1X and 1F97. The combined superposition of these three structures was used as the MR search probe. The use of this probe allowed unambiguous determination of the positions of two of the molecules of β3 Ig domain in the asymmetric unit. The translation function Z-score value for this solution was 10.7. The position of the third molecule could not be identified clearly at this stage. The crystallographic refinement and automatic model building of the MR solution obtained were performed using the PHENIX software suite; the coordinates of only the 2X1X portion of the MR probe were used in calculations. These calculations caused a significant drop in *R*_cryst_/*R*_free_ values by more than 12% each, reaching values of 31.0 and 35.7%, respectively, thus indicating the correctness of the MR solution obtained for the two molecules of the β3 Ig domain. Re-running the MR calculations using one of the refined molecules from the previous step as the MR search probe identified the position of the third molecule.

The next round of refinement and auto-building calculations dropped the *R*_cryst_/*R*_free_ values even further to 25.1 and 28.4%, respectively. The model was then subjected to several rounds of alternating manual rebuilding performed in molecular graphics software suite COOT (23) and crystallographic refinement calculations with either PHENIX or REFMAC software (24). The NCS restraints were used at the initial stages of the refinement except for the last two rounds of refinement. The *R*_cryst_ and *R*_free_ converged to the values of 20.2 and 23.8%, respectively. The structural validation was also performed on the final model; the crystallographic statistics are shown in Table 1. Examination of the trimer interface was performed using PISA (25), and the results are shown in Tables 2 and 3.

**TABLE 1:** **Crystallographic data collection and refinement statistics**

**Data collection**	
Radiation source	Diamond, I04
Wavelength	0.9795 Å
Space group	P3_1_21
Cell dimensions	
*a*, *b*, *c*	81.47, 81.47, 114.26 Å
α, β, γ	90.0, 90.0, 120.0°
Resolution	30 to 2.50 (2.64 to 2.50) Å*^a^*
*R*_sym_*^b^*	7.6% (63.3%)
*R*_p.i.m._*^c^*	3.5% (29.1%)
〈*I*/σ(*I*)〉	13.8 (2.8)
Completeness	99.8% (100.0%)
Redundancy	5.5 (5.7)
No. of unique reflections	15,675

**Refinement**	
Resolution	30.02 to 2.50 Å
No. of reflections used	
Total	15,643
*R*_free_ set	1,560
*R*_cryst_*^d^*/*R*_free_*^e^*	20.2%/23.8%
No. of non-hydrogen atoms in asymmetric unit	
Protein atoms	2636
Solvent atoms	117
*B*-factor (Å^2^)	
Average	43.0
Wilson	52.6
Ramachandran plot analysis, no. of residues in	
Favored regions	94.6%
Allowed regions	5.4%
Disallowed regions	0.0%
Root mean square deviations	
Bond lengths	0.008 Å
Bond angle	1.245°

*^a^* The statistics shown in parentheses are for the highest resolution shell.

*^b^ R*_sym_ = (Σ*_hkl_*Σ*_i_*|*I_i_*(*hkl*) − *I*_mean_(*hkl*)|)/Σ*_hkl_*Σ*_i_I_i_*(*hkl*).

*^c^* Precision-indicating merging *R* factor: *R*_p.i.m._ = (Σ*_hkl_* (1/(*N*-1))^1/2^ Σ*_i_*|*I_i_*(*hkl*) − *I*_mean_(*hkl*)|)/Σ*_hkl_* Σ*_i_I_i_*(*hkl*), where *N* is redundancy.

*^d^ R*_cryst_ = Σ*_hkl_*‖*F*_obs_(*hkl*)| − |*F*_calc_(*hkl*)‖/Σ*_hkl_*|*F*_obs_(*hkl*)|.

*^e^ R*_free_ is the same as *R*_cryst_ for a random subset not included in the refinement of 10% of total reflection.

**TABLE 2:** **PISA interfaces in the crystal structure of Na_v_ channel β3 subunit Ig domain trimer** The following abbreviations are used: *N*_at_, number of atoms from specified molecule that take part in the interface; *N*_res_, number of residues from the specified molecule that take part in the interface; interface area (Å^2^), calculated as one-half of the difference between the total accessible surface areas of the isolated and interfacing structures; Δ*^i^G,* calculated solvation free energy gain upon formation of the interface; Δ*^i^G p* value, probabilistic measure of randomness for the calculated solvation free energy gain; *N*_HB_, number of potential hydrogen bonds across the interface. Contribution to free energy is approximately 0.5 kcal/mol per bond; *N*_SB,_ number of potential salt bridges across the interface. Contribution to free energy is approximately 0.3 kcal/mol per bond.

Molecule 1				Molecule 2				Interface area	Δ*^i^G*	Δ*^i^G*, *p* value	*N*_HB_	*N*_SB_
Chain ID	*N*_at_	*N*_res_	Surface	Chain ID	*N*_at_	*N*_res_	Surface					
			Å^2^				Å^2^	Å^2^	*kcal/mol*			
A	42	13	6244	B	46	15	6759	415.3	−4.6	0.264	0	1
B	44	14	6484	C	46	14	6244	447.5	−3.3	0.268	0	1
C	42	14	6484	A	43	13	6759	420.0	−4.3	0.215	0	1

**TABLE 3:**
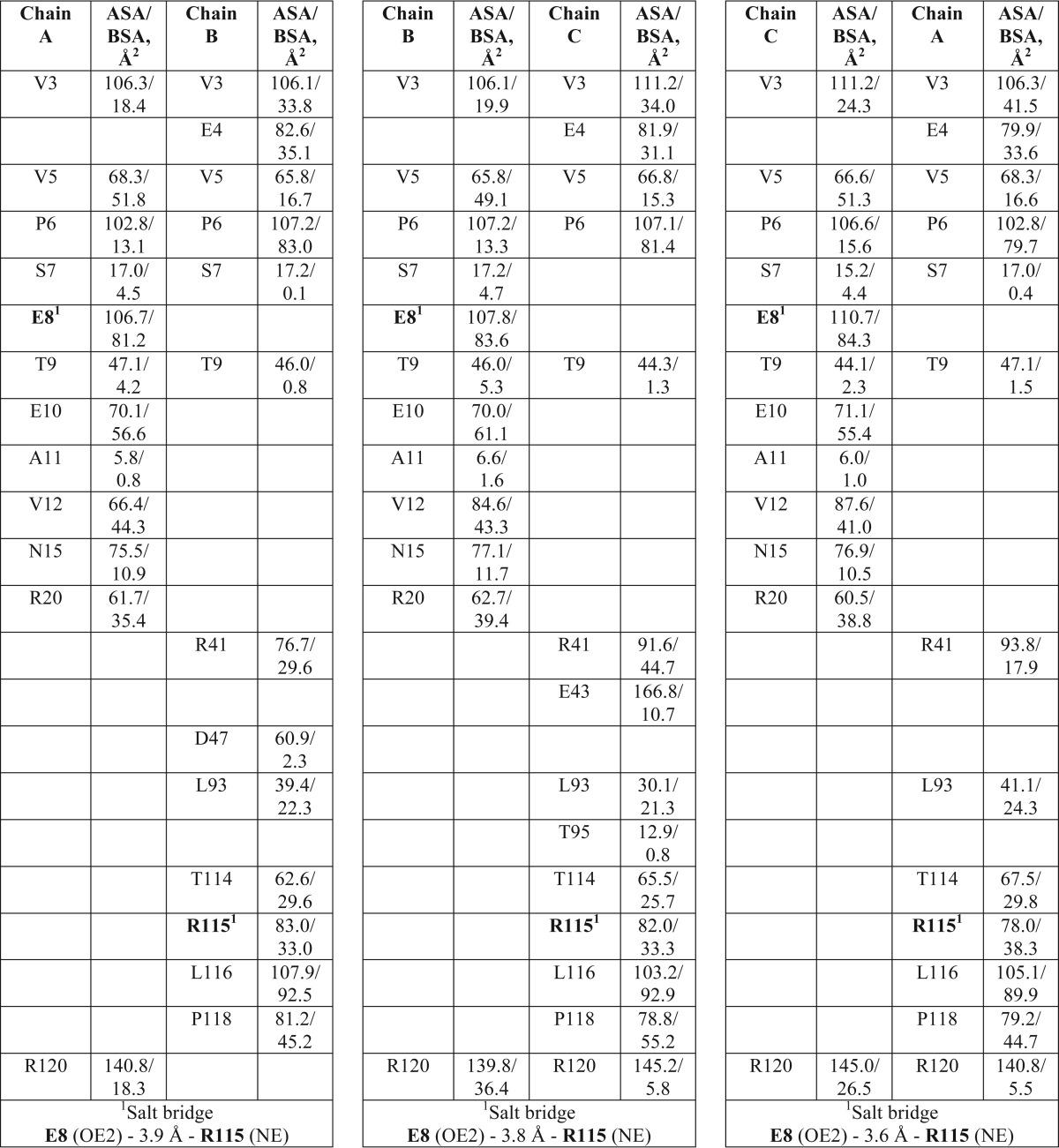
**List of interfacing residues in the crystal structure of Na_v_ channel β3 subunit Ig domain trimer** ASA is accessible surface area; BSA is buried surface area.

##### Analytical Ultracentrifugation (AUC)

Sedimentation velocity experiments were conducted with an Optima XLI (Beckman Coulter) centrifuge using an An60 Ti four-hole rotor. Standard double-sector Epon centerpieces equipped with sapphire windows contained 400 μl of cross-linked β3 Ig domain at 0.5 mg/ml. Interference data were acquired in the continuous mode at time intervals of 315 s and rotor speed of 40,000 rpm, at a temperature of 20 °C with systematic noise subtracted, without averaging and with radial increments of 0.003 cm. The density and viscosity of the buffer and the partial specific volume of the β3 Ig domain were calculated using Sednterp (26). Multicomponent sedimentation coefficient distributions were modeled using Sedfit (27).

##### Atomic Force Microscopy (AFM)

tsA 201 cells were grown in Dulbecco's modified Eagle's medium (Sigma) supplemented with 10% (v/v) fetal bovine serum, 100 units/ml penicillin, and 100 μg/ml streptomycin in an atmosphere of 5% CO_2_/air. Full-length human β3 subunit with a carboxyl-terminal Myc epitope tag was cloned in the vector pFastBacMam (12). Full-length C24A β3 mutant was generated by replacing the carboxyl-terminal enhanced GFP tag (10) with a Myc epitope tag using restriction enzyme KpnI. Correct in-frame insertion was verified by sequencing, and the construct was subcloned into the vector pFastBacMam. Human Na_v_ 1.5 α subunit with a carboxyl-terminal hemagglutinin (HA) epitope tag in the vector pcDNA3N was kindly provided by Dr. C. Valdivia (University of Michigan) (28). Transfection was carried out using calcium phosphate precipitation. A total of 250 μg of DNA was used to transfect cells in 5 × 162-cm^2^ culture flasks. For co-transfections, equal amounts of DNA for each construct were used, up to a total of 250 μg. After transfection, cells were incubated for 48 h at 37 °C to allow protein expression.

All immunoisolation steps were carried out at 4 °C. Transfected cells were solubilized in a buffer containing 1% (v/v) Triton X-100, 25 mm Tris-HCl, pH 7.5, 150 mm NaCl, 10 mm EDTA, 1 mm PMSF, and protease inhibitor mixture (Complete, Roche Applied Science) for 1 h, before centrifugation at 62,000 × *g* to remove insoluble material. The solubilized extracts were incubated with anti-Myc- or anti-HA-conjugated agarose beads (Sigma) for 3 h. The beads were washed extensively with the above buffer without protease inhibitors, and bound proteins were eluted with either Myc or HA peptide (100 μg/ml in the same buffer). Samples were analyzed by SDS-PAGE, followed by silver staining and/or immunoblotting, using either mouse monoclonal anti-Myc (Invitrogen, R950-25) or mouse monoclonal anti-HA (Covance, HA.11 clone 16B12, MMS-101P) primary antibodies, followed by horseradish peroxidase-conjugated goat anti-mouse antibodies (Bio-Rad). Immunopositive bands were visualized using enhanced chemiluminescence.

Isolated protein samples were diluted to a final concentration of ∼40 pm, and 45 μl of the sample was allowed to adsorb to freshly cleaved mica disks. After a 5-min incubation, the sample was washed with Biotechnology Performance Certified-grade water (Sigma) and dried under nitrogen. Imaging was performed with a Veeco Digital Instruments Multimode AFM controlled by a Nanoscope IIIa controller. Samples were imaged in air, using tapping mode. The silicon cantilevers used had a drive frequency ∼300 kHz and a specified spring constant of 40 newtons/m (Olympus). The applied imaging force was kept as low as possible (*A*_s_/*A*_0_ ∼0.85).

For particles within complexes, particle heights and diameters were measured manually using the Nanoscope software and used to calculate molecular volumes, according to Equation 1,

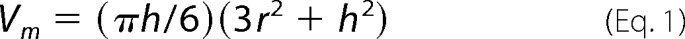
 where *h* is the particle height, and *r* is the radius (29). Equation 1 assumes that the adsorbed particles adopt the form of a spherical cap.

Molecular volume based on molecular mass was calculated using Equation 2,

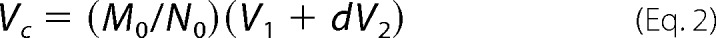
 where *M*_0_ is the molecular mass; *N*_0_ is Avogadro's number; *V*_1_ and *V*_2_ are the partial specific volumes of particle (0.74 cm^3^/g) and water (1 cm^3^/g), respectively; and *d* is the extent of protein hydration (taken as 0.4 g of water/g of protein). Note that we have previously compared volumes, measured as described above, with predicted volumes for various proteins over a wide range of molecular masses, and we found that measured and predicted volumes corresponded closely (28, 30, 31). Hence, we are confident that molecular volumes are determined accurately by our AFM imaging.

Histograms were drawn with bin widths chosen according to Scott's Equation 3,

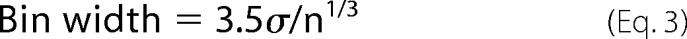
 where σ is an estimate of the standard deviation, and *n* is the sample size (32). Where Gaussian curves were fitted to the data, the number of curves was chosen so as to maximize the *r*^2^ value while giving significantly different means using Welch's *t* test for unequal sample sizes and unequal variances (33).

##### Fluorescence Photoactivated Localization Microscopy (FPALM)

The hASIC-mEos2 construct was a gift from Prof. R. H. Chow (University of Southern California). The β3-mEos2 construct was made by exchanging pEGFP (10) for mEos2 in the β3-EGFP construct, using AgeI and NotI restriction sites. Correct in-frame insertion was verified by sequencing. Coverslips (18-mm, Karl Hecht GmbH) were cleaned with 1 m hydrochloric acid for 12 h, followed by washing with distilled water. After a 12-h treatment with acetone and distilled water, coverslips were stored in 100% ethanol until use. Sterile coverslips were placed in 35-mm tissue culture dishes and coated with Matrigel (1:50 in modified Eagle's medium, pH 7.4; BD Biosciences). HEK293 cells, grown on these coverslips, were transiently transfected 48 h after plating with Lipofectamine 2000 (Invitrogen). 24 h after transfection, cells were washed twice with phosphate-buffered saline (PBS), fixed with 4% paraformaldehyde, 0.2% glutaraldehyde (SERVA Electrophoresis GmbH) in PBS for 15 min at 4 °C, followed by 30 min at room temperature. Quenching was done with filter-sterilized NH_4_Cl in Ringer's solution, pH 7.4, for 15 min, and cells were finally washed three times with PBS.

Total internal reflection fluorescence (TIRF) photoactivation localization microscopy (PALM) imaging was performed on a Nikon Ti Eclipse equipped with fiber-coupled lasers of different wavelengths as follows: 405 nm (60-milliwatt Phoxx, Omicron), 491 nm (100-milliwatt Calypso, Cobolt) and 561 nm (100-milliwatt Jive, Cobolt). Fluorescence emission was detected using a 100× objective (Nikon Apo TIRF 1.49 NA) together with an electron-multiplying charge-coupled device camera (Andor Technology). 20,000 Images of 82 × 82-μm area were collected with an exposure time of 100 ms. The mEos fluorophore was activated with the 405-nm laser at very low intensities (microwatts) and excited with the 561-nm laser with the intensity set to maximum. Peaks were localized using an algorithm for PALM analysis provided by (34) and fitted by a cylindrically symmetric Gaussian point spread function. The average localization precision ranged from 18 to 25 nm, and α (average number of appearances of individual molecules, *i.e.* blinking) ranged from 2 to 3 per mEos2 molecule for the mEos2 fusion constructs used here. Subsequent analysis to calculate the pair correlation function of the steady-state distribution of β3 was carried out according to Refs. 35, 36, using code written in MATLAB (MathWorks).

## RESULTS

### 

#### 

##### Structure of the Na_v_ β3 Subunit Ig Domain

**FIGURE 1.**
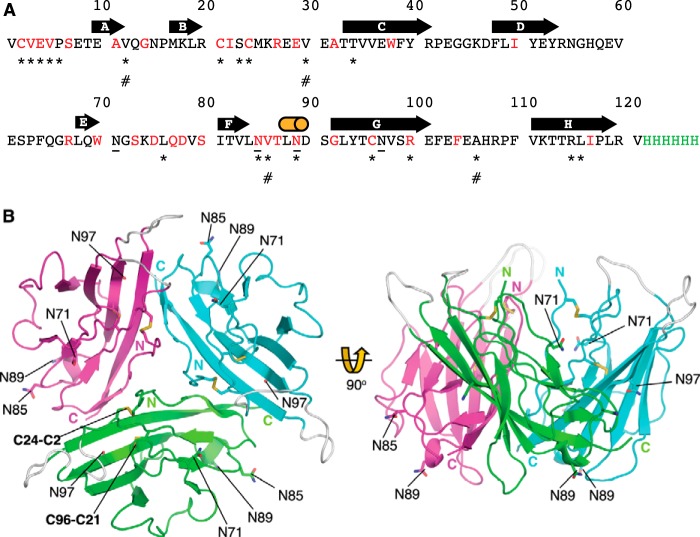
**Sequence and structure of the β3 Ig domain.** *A*, primary sequence of the β3 Ig domain cDNA construct used in the study. The amino acid residues that are fully conserved between β3 and β1 are indicated in *red*. The hexa-His tag used for protein purification is indicated in *green*. Individual elements of β sheet are indicated by a *bold arrow*, and the 3_10_ helix is indicated by a *cylinder*. Potential *N*-linked glycosylation sites are *underlined.* Amino acid residues mentioned in the main text are indicated by *. Amino acid residues mutated in the cardiac conduction diseases and mentioned in the text are indicated by #. *B*, crystal structure of Na_v_ β3 subunit Ig domain showing trimeric arrangement of molecules. Schematic representation of the Na_v_ β3 subunit Ig domain molecules present within the crystal's asymmetric unit. Amino and carboxyl termini are labeled. The Cys-21–96 disulfide bond and the Cys-2–24 disulfide bond are labeled on the *green* protomer. Potential *N*-linked glycosylation sites Asn-71, Asn-85, Asn-89, and Asn-97 are shown as *sticks* and labeled. Loops that are not visible in the electron density maps due to local disorder are shown at their plausible theoretical positions in *gray*.

The β3 subunit Ig domain protein was purified from the supernatant of transfected HEK293F cells. Under these conditions, the ER targeting signal was efficiently removed, and the disulfide bonds were formed correctly (13). To enable crystallization, carbohydrate side chains were removed by peptide:*N*-glycosidase F digestion. The structure was determined to a resolution of 2.5 Å, with phases obtained using the molecular replacement method. The primary sequence of the β3 Ig domain protein used in these experiments is shown in Fig. 1*A.* It should be noted that for consistency with prior convention (11, 37–39), the amino acids are numbered from the first residue of the mature protein (*i.e.* lacking the ER targeting signal). The β3 Ig domain assembled as a trimer in the asymmetric unit (Fig. 1*B*). The β3 subunit protomers in the asymmetric unit are related to each other by a pseudo 3-fold NCS axis (Tables 1–3). The rotation around the pseudo 3-fold NCS axis between protomers C and B is 117.1°, between protomers A and C is 120.8°, and between protomers B and A is 122.2°. This results in different buried surface areas between the β3 Ig domain protomers within the trimer. The buried surface area between C and B is 895.0 Å^2^, between A and B is 839.9 Å^2^, and between B and A is 830.5 Å^2^. The total buried surface area within the trimer is around 2565 Å^2^. Potential *N*-linked glycosylation sites of each protomer point away from the trimer interface (Fig. 1*B*). Hence, any sugar residues added *in vivo* are unlikely to prevent trimer assembly.

**FIGURE 2.**
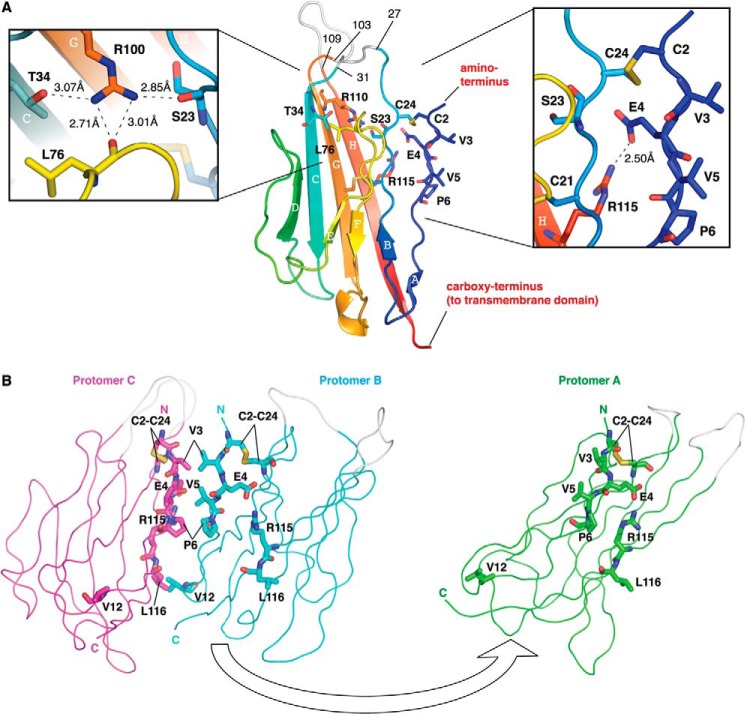
**β3 Ig domain protomer and trimer interface.** *A,* schematic representation of a single Na_v_ β3 subunit Ig domain protomer. The polypeptide chain is colored in gradient from amino terminus (*blue*) to carboxy terminus (*red*). The β-sheets are labeled in alphabetical order. Residues 27–31 and 103–109 correspond to loops not visible in the electron density maps due to local disorder; they are shown at their plausible theoretical positions in *gray* and labeled. The Cys-2–24 disulfide bond is shown as *sticks* and labeled. A close-up of hydrogen bonding interactions of residue Arg-100 with Ser-23, Thr-34, and Leu-76 is shown on the *left*. A close-up of the region surrounding the Cys-2–24 disulfide bond, including the Val-3, Val-5, and Pro-6 residues is shown on the *right*. The lengths of hydrogen bonds are indicated. *B*, location of the key trimer interface residues. For clarity, protomer A (*green*) is removed from the rest of the trimers by application of 180° rotation around its center of mass and rightward translation. *Protomers B* (*cyan*) and *C* (*magenta*) are in the same orientation as shown in Fig. 1*B*. The interfacing residues discussed in the text are shown as *sticks* and labeled.

**FIGURE 3.**
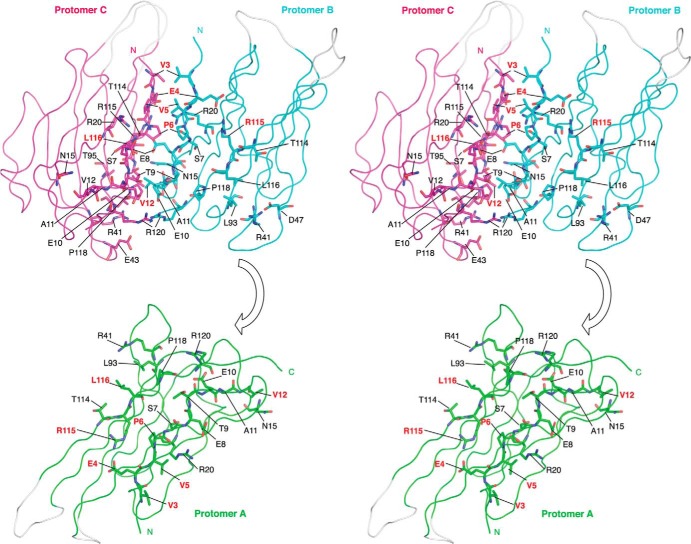
**Wide-eyed stereo picture of the β3 subunit Ig domains.** The trimeric arrangement of protomers is shown in ribbon representation. The interfacing residues in all three protomers are shown as *sticks* and labeled. Amino acid residues discussed in the main text are shown in *red*. Amino acid residues shown in Table 3 are shown in *black*. To demonstrate more clearly the interfacing residues, *protomer A* (*green*) is removed from the rest of the trimer by application of 180° rotation around its center of mass and translating it downward. *Protomers B* (*cyan*) and *C* (*magenta*) are in the same orientation as shown in Fig. 2*B*. The β3 subunit Ig domain protomers in the asymmetric unit are related to each other by pseudo 3-fold NCS axis.

Key features common to all Ig domains are present in the protomer. These include a buried disulfide bond (Cys-21–96) that stabilizes adjacent faces of the molecule and the presence of Ig domain-typical β-sheets (40). However, the protomer also exhibits some structural features that are unusual for Ig domains. In particular, the first 10 amino-terminal residues of Ig domains are normally stabilized by intramolecular antiparallel β-sheets (40). This secondary structure element is missing in β3. Instead, the region is held in place by a surface intramolecular disulfide bond, Cys-2–24. An intramolecular salt bridge between Glu-4 and Arg-115 further constrains the local conformation of the peptide chain (Fig. 2*A*). As a result, the side chains of residues Val-3, Val-5, and Pro-6 are surface-exposed. These residues from each protomer provide the main trimer-stabilizing hydrophobic contacts. Further stability in this region may be provided by residue Arg-100, whose side chain points into the Ig domain core and forms hydrogen bonds with the side chain of residue Thr-34, and the main chain carbonyls of residues Ser-23 and Leu-76 (Fig. 2*A*). The trimer-stabilizing interface residues shared by all protomers are shown in Fig. 2*B*. An intersubunit hydrophobic interaction between Leu-116 and Val-12 from adjacent protomers that lies outside of the trimer interface also contributes to trimer stability (Fig. 2*B*). Some additional residues contribute by making hydrogen bonding networks through water molecules to the main chain but not in all protomer interfaces (Tables 2 and 3). A wide-eyed stereo image of the trimer interface region that highlights all the residues noted in Table 3 is shown in Fig. 3.

##### Purified Na_v_ β3 Ig Domains and Full-length β3 Subunits Form Monomers, Dimers, and Trimers

**FIGURE 4.**
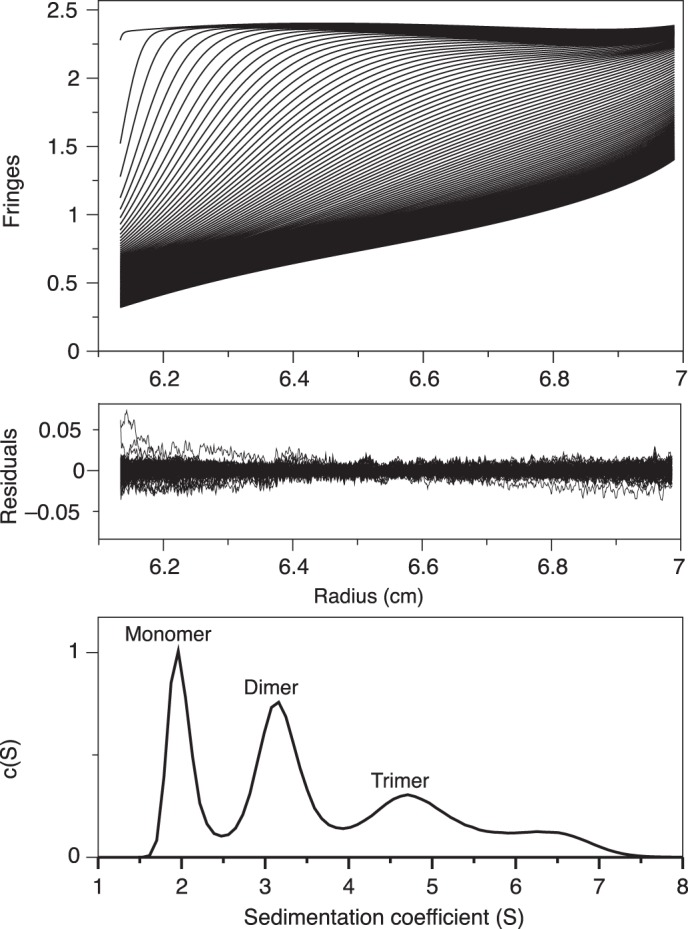
**Na_v_ β3 subunit Ig domain oligomerizes in solution.** Analytical ultracentrifugation sedimentation velocity data of cross-linked β3 Ig domain (see under “Experimental Procedures”). The residuals are from the fit with the hybrid discrete/continuous model. Component sedimentation coefficient distribution showing populations of monomeric, dimeric, trimeric (and larger) species with a uniform frictional ratio of *F_k,w_* = 1.4. The final r.m.s.d. was 0.006.

We used AUC to assess the presence of β3 Ig domain oligomers in solution. At a concentration of 0.5 mg/ml in a chemically cross-linked sample, we identified monomers, dimers, and trimers (Fig. 4). These data confirm that the purified β3 Ig domains, alone and free in solution, can interact homophilically.

**FIGURE 5.**
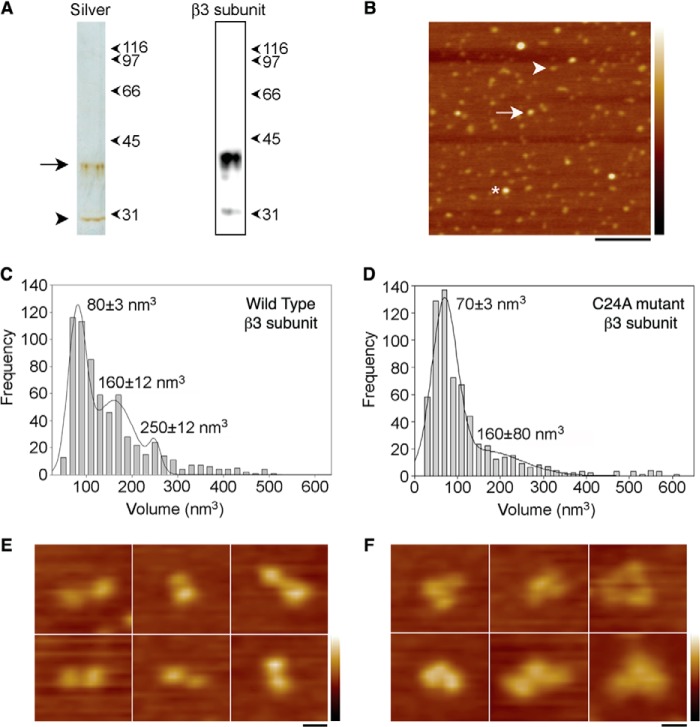
**Assembly state of the β3 subunit as determined by AFM imaging.** *A*, analysis of immunoisolated Myc-tagged β3 subunits by SDS-PAGE, followed by either silver staining (*left panel*) or immunoblotting using an anti-Myc antibody (*right panel*). Full-length β3 subunit is visible on the silver-stained gel as an ∼40-kDa band (*arrow*), typical of glycosylated β3 (13). A band at ∼30 kDa (*arrowhead*) likely represents the unglycosylated form of the protein. The same two bands are seen on the immunoblot. *B*, low magnification AFM image of immunoisolated β3 subunits. Particles indicated by the *arrow* and the *asterisk* are approximately two and three times, respectively, the size of the particle indicated by the *arrowhead. Scale bar,* 200 nm; height scale, 0–4 nm. *C*, frequency distribution of molecular volumes of the β3 subunit particles. *D*, frequency distribution of molecular volumes of the C24A β3 mutant subunit particles. The curves in *C* and *D* indicate the fitted Gaussian functions. The peaks of the distribution (±S.E.) are indicated. *E*, zoomed images of splayed dimers of C24A mutant β3 subunits. *F*, zoomed images of splayed trimers of C24A mutant β3 subunits. *Scale bar,* 20 nm; height scale, 0–3 nm.

The assembly state of a purified, full-length β3 subunit was investigated by single molecule resolution imaging using AFM. The carboxyl-terminal Myc-tagged β3 subunit was immunoisolated from a detergent extract of transfected tsA 201 cells. Analysis of the isolated protein by SDS-PAGE followed by silver staining (Fig. 5*A*, *left panel*) revealed a band at ∼40 kDa, typical of a mature glycosylated β3 subunit; a band at ∼30 kDa was also present, likely representing the unglycosylated form of the protein. The same two bands were seen on an anti-Myc immunoblot (Fig. 5*A*, *right panel*). A low magnification AFM image of the protein shows a spread of variously sized particles (Fig. 5*B*). The particles indicated by the *arrow* and the *asterisk* in Fig. 5*B* are approximately two and three times, respectively, the size of the particle indicated by the *arrowhead*. The dimensions of a number of β3 subunit particles were measured, and particle volumes were calculated using Equation 1 (see under “Experimental Procedures”). A frequency distribution of molecular volumes (Fig. 5*C*) had three peaks as follows: at 80 ± 3 nm^3^, 160 ± 12 nm^3^, and 250 ± 12 nm^3^ (*n* = 650). The expected volume of a protein with a molecular mass of 40 kDa, calculated using Equation 2 (see under “Experimental Procedures”), is 76 nm^3^, very close the volume of the first peak, indicating that the three peaks represent β3 subunit monomers, dimers, and trimers. The three differently sized particles highlighted in Fig. 5*B* belong to these three peaks.

##### Mutational Cleavage of theCys-2–24 Disulfide Bond Destabilizes β3 Subunit Oligomer Assembly

Our structure indicates a critical role for the Cys-2–24 disulfide bond in maintaining the orientation of the trimer interface (Fig. 2*A*). We have previously shown that a C24A mutant of full-length β3 failed to bind the recombinant wild-type β3 Ig domain, as detected by immunoprecipitation (13). We therefore investigated the oligomeric status of the full-length C24A mutant using AFM. When the C24A mutant β3 subunit was isolated and subjected to the same analysis as the wild-type β3 protein, the frequency distribution of molecular volumes had only two peaks, at 70 ± 3 and at 160 ± 80 nm^3^ (*n* = 500), corresponding to monomers and dimers. The dimer peak was reduced compared with wild-type β3 (Fig. 5*D*). Higher resolution AFM images from both the wild-type and C24A mutant revealed examples of particles with “splayed-out” conformations, corresponding to doublets and triplets of particles. These likely represent β3 subunit complexes that have partially dissociated after attachment to the mica substrate. Such images provide direct visual evidence for the presence of full-length β3 dimers and trimers. Significantly, these partially dissociated β3 subunits were more common with the C24A mutant than the wild-type protein (Table 4). Representative images are shown for the C24A mutant in Fig. 5, *E* (dimers) and *F* (trimer). Together with the altered frequency distribution, these results indicate a reduced stability of the oligomeric structures formed by the C24A mutant β3 subunit.

**TABLE 4:** **The C24A mutant destabilizes β3 oligomers** Numbers of images counted and of splayed dimers and trimers exhibited by the β3 wild-type and β3 C24A mutant.

	Wild-type β3	C24A mutant β3
Total no. of images	65	65
Splayed dimers	16	40
Splayed trimers	2	11

##### Na_v_ β3 Subunit Forms Trimers on the Plasma Membrane

**FIGURE 6.**
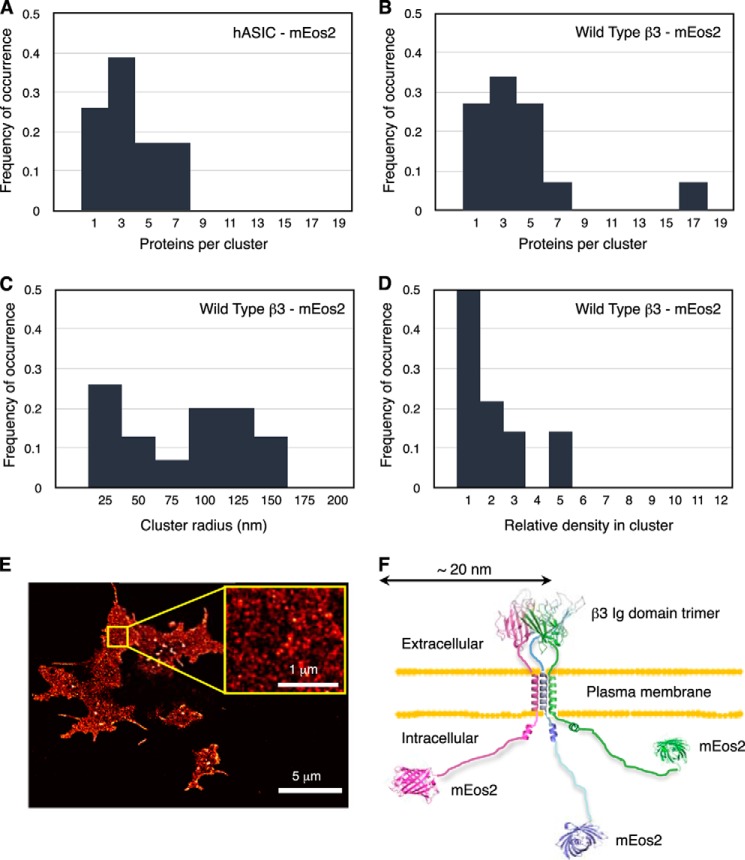
**Full-length Na_v_ β3 subunit forms trimeric clusters *in vivo*.** The frequencies plotted in Fig. 5, *A–D,* represent the distributions of the respective fit parameters obtained from separate pair correlation analyses of 30 different regions in five cells. The pair correlation (*PC*) function quantifies the probability of finding another molecule at a certain distance away from a molecule compared with the expectation for a random molecule distribution. From the amplitude and shape of this deviation of the pair correlation curve from a random distribution, a number of parameters are estimated, such as cluster radius, number of molecules per cluster, and relative density in cluster. *A–D,* distributions of cluster parameters for hASIC-mEOS2 (acid-sensing ion channel) and full-length β3-mEOS2 subunit estimated by pair correlation analysis plotted for proteins per cluster (*A* and *B*), cluster radii (*C*), relative protein density in cluster (*D*). *E*, TIRF-PALM image showing the spatial distribution of β3-mEos2 expressed in fixed HEK293 cells. The β3-mEos2 molecules are found as small, dense clusters. To reconstruct this distribution of β3-mEos2, 119,138 molecules are plotted as two-dimensional Gaussian spots with a localization precision of ∼20 nm. The expanded area (*yellow box*) shows a typical region of the cell with a homogeneous distribution (no holes or big aggregates) used for pair correlation analysis. *F*, schematic showing the approximate dimensions expected for the full-length β3 trimer on the plasma membrane. The stalk connecting the Ig domains to the transmembrane domain is likely to be extended and unstructured (11). The arrangements and angles adopted by the transmembrane domains are currently uncertain. Based on its NMR-derived structure, the intracellular domain was modeled as disordered, with a short region of juxtamembrane α helix (12). The mEos2 label was modeled using the Protein Data Bank code 3S05.

To investigate the spatial distribution of full-length β3 subunits on the surface of HEK293 cells, we used FPALM analysis (41, 42). This super-resolution imaging technique requires fluorescent probes that either photoswitch or photoconvert. The full-length β3 subunit was tagged at the carboxyl terminus with the monomeric green-to-red photoconvertable protein mEos2. Individual mEos2 molecules were converted to the red species sparsely but continuously using a 405 nm laser, whereas TIRF imaging was simultaneously performed with a 561 nm laser. With the mEos2 fluorophore, a localization precision of ∼10 nm could be achieved (43). We used pair correlation analysis (44) to examine quantitatively the nanoscale organization of these subunits. To confirm the power of pair correlation analysis as applied to our FPALM data, we first examined the plasma membrane distribution of mEos2-labeled acid-sensing ion channel hASIC. This molecule is a known trimer (45). Analysis of 30 separate regions on five cells, each with a 1- μm^2^ area, generated a size distribution with a peak frequency consistent with a trimeric channel (Fig. 6*A*). When the same analysis was repeated using β3-mEos2 molecules, a similar distribution with identical peak frequency was obtained (Fig. 6*B*). The spreads of the distributions reflect the fitting uncertainty. This is mostly due to uncertainty in determining the actual blinking number, *i.e.* the repeated detection of the same protein, and the uncertainty in detecting, *i.e.* photo-activating, all fluorescent proteins in a cluster (44). The distribution spreads, however, are similar for hASIC-mEos2 and β3-mEos2 (Fig. 6, *A* and *B*). Thus, we conclude that full-length β3 subunits on the plasma membrane adopt a predominantly trimeric structure. Using pair correlation analysis, we further determined that β3 subunits were organized into clusters of radii <50 nm (Fig. 6*C*), although larger sized clusters of β3 were also detected that may indicate higher order assemblies. The relative density of the protein within the clusters (ϕ^(cluster)^) (44) is low (Fig. 6*D*). This suggests that the mEos2 fluorescent tag is relatively unconstrained. Fig. 6*E* shows a typical TIRF-PALM image in fixed HEK293 cells, used to reconstruct the distribution of β3-mEos2. Fig. 6*F* presents a model of the full-length mEos2-tagged β3 molecule. The 32-amino acid intracellular region of β3 is known to be largely disordered (12); it could therefore extend about 17 nm. The mEos2 tag is about 4 nm long (46). Hence, the β3 trimer, with a mEos2 tag at the carboxyl terminus, could be up to 21 nm in radius. This is consistent with the FPALM data.

##### Complex Binding of β3 Subunits to Na_v_ 1.5 α Subunits

**FIGURE 7.**
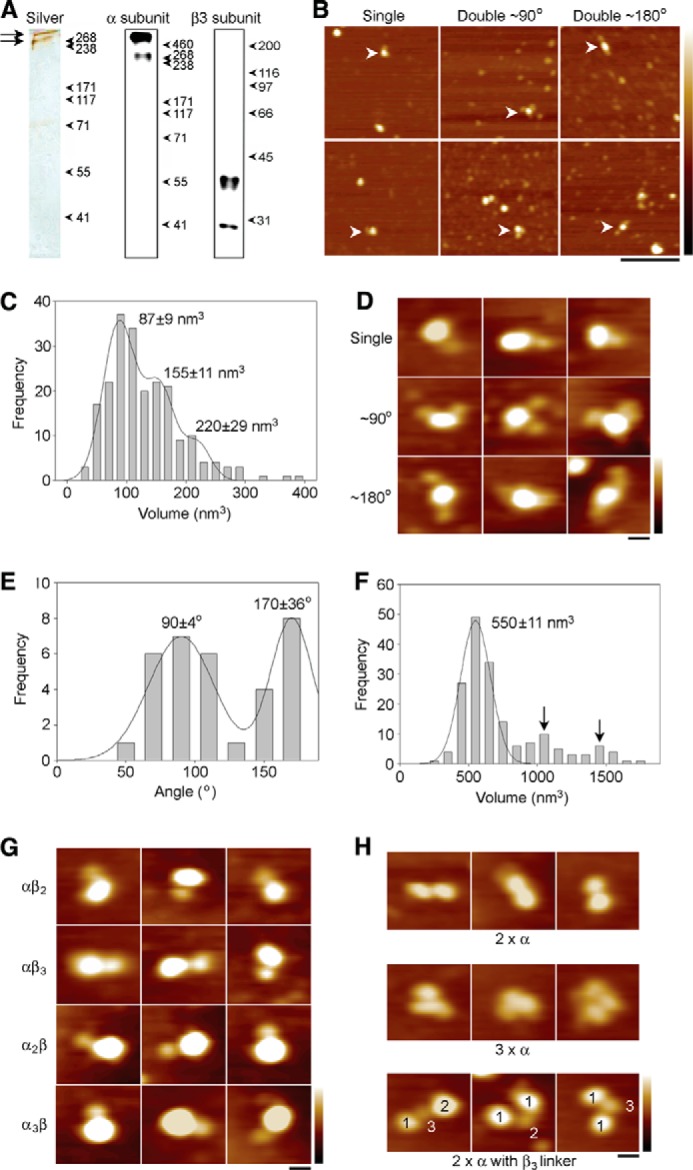
**Interaction between the Na_v_ 1.5 α subunit and the β3 subunit, as determined by AFM imaging.** *A*, proteins were isolated by immunoaffinity chromatography, using the carboxyl-terminal HA tag on the α subunit. Immunoisolated samples were analyzed by SDS-PAGE, followed by either silver-staining (*left panel*) or immunoblotting using anti-HA (*center panel*) or anti-Myc (*right panel*) antibodies. The silver-stained gel shows two bands at ∼250 and >500 kDa (*arrows*). The same two bands are seen on the immunoblot and likely correspond to the α subunit monomer and an oligomer/aggregate, respectively. The anti-Myc blot shows the same two bands seen when the β3 subunit was expressed alone. Molecular mass markers (kDa) are indicated. *B*, low magnification AFM images of a sample containing co-isolated Na_v_ α/β3 subunits. *Arrowheads* indicate α subunits singly decorated by β3 subunits (*left panels*) or doubly decorated at either ∼90° (*center panels*) or ∼180° (*right panels*). *Scale bar,* 200 nm; height scale, 0–5 nm. *C*, frequency distribution of volumes of peripheral particles bound to large central particles. The *curve* indicates the fitted Gaussian functions. The peaks of the distributions are indicated and are consistent with β3 monomers, dimers, and trimers. *D*, gallery of zoomed images showing singly decorated α subunits or α subunits doubly decorated at either ∼90 or ∼180°, as indicated. *Scale bar,* 20 nm; height scale, 0–3 nm. *E*, frequency distribution of angles between pairs of bound β3 subunits. The curve indicates the fitted Gaussian functions. The peaks of the distribution are indicated. *F*, frequency distribution of volumes of the large decorated central particles for those structures with visible α and β3 subunits. The *curve* indicates the fitted Gaussian function. The peak of the distribution is indicated. The *two arrows* indicate potential peaks at two and three times the volume of the main peak. *G*, examples of decoration events involving multiples of either α subunits or β3 subunits, as indicated. *Scale bar,* 20 nm; height scale, 0–3 nm. *H*, examples of double (*upper panels*) and triple (*center panels*) α subunits and of α subunits connected by β3 subunit oligomers (*lower panels*). The *numbers* in the *lower panels* indicate the stoichiometries of the various particles. *Scale bar,* 20 nm; height scale, 0–3 nm.

A clear implication from our structure is that β3 subunits may be capable of cross-linking Na_v_ α subunits. To investigate this possibility, we again used AFM imaging. We examined the major heart Na_v_ α subunit Na_v_ 1.5, bearing an HA carboxyl-terminal epitope tag, with and without Myc-tagged wild-type β3, and Myc-tagged C24A β3 mutant. We used this α subunit because β3 is known to interact with Na_v_ 1.5 in the heart and modify its gating properties (10, 47), and the *Scn3b* gene deletion in the mouse leads to specific cardiopathologies (14, 15). The α subunit was isolated from a detergent extract of the co-transfected cells by immunoaffinity chromatography, using the HA tag. Analysis of the isolated protein by SDS-PAGE, followed by silver staining (Fig. 7*A*, *left panel*), revealed two bands at ∼250 and >500 kDa (*arrows*). The same two bands were seen on an anti-HA immunoblot (Fig. 7*A*, *center panel*) and likely correspond to the Na_v_ 1.5 α subunit monomer and an oligomer/aggregate, respectively. The anti-Myc blot (Fig. 7*A*, *right panel*) shows the same two bands seen when the β3 subunit was expressed alone (Fig. 5*A*). This result demonstrates that the α and β3 subunits can be co-isolated from co-transfected cells; however, the association between them is relatively weak, because the β3 subunit was not visible on the silver stain.

The association between Na_v_1.5 α and the β3 subunit was evident on the AFM images, which showed some examples of large central particles decorated by one or more smaller peripheral particles (Fig. 7*B*). For these images, a frequency distribution of the volumes of the peripheral particles (Fig. 7*C*) had three peaks, at volumes of 87 ± 9, 155 ± 11, and 220 ± 29 nm^3^, similar to the volumes seen for the β3 subunit alone (Fig. 5*C*). Hence, the α subunits became decorated by a mixture of β3 subunit monomers, dimers, and trimers. Of a total of 900 large particles counted, 113 (12.5%) were decorated by one additional small particle, and 31 (3.4%) were decorated by two additional small particles. In contrast, when protein was isolated from cells expressing the α subunit alone, 40/1006 (3.9%) of the large particles were singly decorated by small particles, and 3/1006 (0.3%) were doubly decorated. Hence, the majority of decoration events seen when the α and β3 subunits were co-isolated represented specific α/β3 interactions. Fig. 7*D* shows a gallery of zoomed images of Na_v_ 1.5 α subunits decorated by either one (*upper panels*) or two β3 subunits (*center* and *lower panels*). Double decoration of the α subunit occurred at angles of either ∼90° (Fig. 7*D, center panels*) or ∼180° (*lower panels*). Consistent with this observation, a frequency distribution of angles between pairs of bound β3 subunits (Fig. 7*E*) had two peaks, at 90 ± 4 and 170 ± 36° (*n* = 33). The ratio of the numbers of particles in the two peaks was 21:12 (*i.e.* 1.7:1). This result, with decoration at ∼90° and ∼180° in a ratio of ∼2:1, indicates that β3 subunits bind Na_v_ 1.5 α subunits with 4-fold symmetry.

A 1:4 binding stoichiometry between the α and β3 subunits, coupled with the ability of the β3 subunit to dimerize and trimerize, would provide a mechanism for producing large clusters of α and β3 subunits on the plasma membrane. For those images with visible α and β3 subunits, a frequency distribution of volumes of the decorated α subunits (Fig. 7*F*) had a single major peak at 550 ± 11 nm^3^, close to the expected volume for a protein of molecular mass 250 kDa (475 nm^3^). Although there were no clear higher peaks in the distribution, there was some indication of decorated particles in the volume ranges ∼1000 and ∼1500 nm^3^ (*arrows,* Fig. 7*F*), which may represent a small number of α subunit dimers and trimers. There is evidence for this clustering in our AFM images. For instance, Fig. 7*G* shows a gallery of zoomed images of complexes of various stoichiometries between α and β3 subunits. In addition, Fig. 7*H* shows examples of dimers (*upper panels*) and trimers (*center panels*) of α subunits and also of pairs of α subunit particles apparently held together by β3 subunit oligomers (*lower panels*). Note that only a minority of the larger α subunit-sized particles was visibly decorated by peripheral particles. However, an analysis of all particles indicated the presence of higher order structures, consistent with α subunit dimers and trimers; these were more common in the presence of the wild-type β3 subunit (Table 5). In the latter case, these structures may represent particles where the associated β3 subunits were buried within the complexes, surrounded by α subunits, and thus not resolved. In contrast, the C24A mutant β3 subunit did not increase the proportion of α subunit dimers and trimers above the background level (Table 5).

**TABLE 5:** **The C24A β3 mutant reduces α subunit cross-linking** Numbers of α subunit monomers, dimers, and trimers isolated from cells transfected with α subunits alone or α subunits with β3 wild-type or with β3 C24A mutant.

	Na_v_1.5 α subunit	Na_v_1.5 α subunit + wild-type β3	Na_v_1.5 α subunit + C24A β3 mutant
Monomers	728	728	728
Dimers	348	722	264
Trimers	340	566	273

## DISCUSSION

The Ig domain of the Na_v_ channel β3 subunit has a surprising structure. To our knowledge, there are no previous reports of natural trimeric Ig domains, and the β3 trimer interface is unlike any other Ig domain binding site. The mixture of monomers, dimers, and trimers observed by AUC (Fig. 4) suggests that the isolated Ig domains interact with relatively low affinity in solution. Similar low affinity interactions have been described for other isolated Ig domains from cell adhesion molecules (48). To detect such interactions at a protein concentration that would avoid nonideality effects in our AUC experiments, we used cross-linking.

As shown by AFM, isolated full-length β3 subunits similarly form monomers, dimers, and trimers (Fig. 5*C*). Again, this suggests that the interactions between individual β3 subunits may be relatively weak, especially when the protein is removed from its normal cellular context. However, when localized to the two-dimensional surface of the plasma membrane, such low affinity interactions can still be significant (48). Indeed, low affinity interactions may be advantageous, because they will facilitate a more dynamic protein network. The FPALM analysis confirmed that full-length β3 molecules on the plasma membrane of HEK293 cells form trimers as a major species (Fig. 6*B*). The FPALM analyses also reported low ϕ^(cluster)^ values (Fig. 6*D*). This finding suggests that the mEos2 tag is relatively unconstrained and is consistent with our previous demonstration that the β3 intracellular region is largely disordered (Fig. 6*F*) (12). Thus, the AUC, AFM, and FPALM analyses confirm that our structure is not a crystal packing artifact. The structure is also consistent with our recent demonstration that β3 subunit Ig domains can interact homophilically when expressed *in vivo* (13).

We have previously predicted the existence of the surface disulfide bond Cys-2–24 in the β3 Ig domain (10, 11), and we have recently confirmed its presence by mass spectrometry (13). The importance of this unusual feature is now clear. Within the protomer, the strong covalent Cys-2–24 disulfide bond is required to constrain the local polypeptide chain, so as to force the unfavorable exposure of residues Val-3, Val-5, and Pro-6 (Fig. 2, *A* and *B*). As a result, these hydrophobic residues become available to form the trimer interface. The C24A mutant lacks this critical surface disulfide bond, and the trimer interface should therefore be weakened. As expected, our AFM data confirmed a reduced oligomer stability in the mutant (Fig. 5*D* and Table 4). However, the presence of “splayed” dimers and trimers that nevertheless remain associated (Fig. 5, *E* and *F*) provides evidence for additional interactions between β3 subunit regions that may lie outside the Ig domain (Fig. 6*F*). Interestingly, the transmembrane domain of β3 contains a highly conserved glutamic acid residue within an otherwise uniformly hydrophobic region (11). Model transmembrane α-helices with a membrane-embedded glutamic acid readily form dimers and trimers, stabilized by hydrogen bonds between the protonated glutamic acid side chains (49). We suggest that this feature may further stabilize β3 dimers and trimers *in vivo*.

The AFM data also show that the Na_v_ 1.5 α subunit can bind β3 subunits at up to four separate sites (Fig. 7, *D* and *E*). This result is consistent with the known presence of four internally homologous domains within the Na_v_ α subunit structure (2). However, in at least some Na_v_ channels, the β3 carboxyl terminus interacts with the single carboxyl terminus of the α subunit (50). Hence, not all of the β3-binding sites on the Na_v_ 1.5 α subunit may be thermodynamically equivalent.

The existence of β3 oligomers, and the presence of multiple β3-binding sites on the α subunit, implies that the β3 subunit could potentially induce higher order Na_v_ channel clustering. AFM particles corresponding to dimeric and trimeric α subunits were about twice as common in cells co-expressing α and β3 subunits, compared with cells expressing α subunits alone (Table 5). Only cells co-expressing α and wild-type β3 subunits generated images where α subunits were visibly connected to each other via particles consistent with β3 trimers (Fig. 7*H*). We note that such large scale structures are unlikely to fully survive the immunoisolation procedure and are also difficult to discriminate from nonspecific aggregates by AFM. We have previously observed similar behavior for other protein complexes (31). For these reasons, we suggest that the observed distribution of α subunit oligomers underestimates the proportions of cross-linked α subunits existing in the intact cell. Significantly, such higher order α subunits were reduced to the background level for α subunits co-expressed in cells with the C24A β3 mutant (Table 5). This result provides additional evidence that β3 subunit oligomers enhance α subunit cross-linking.

In CHO cells, the β3 subunit induced a hyperpolarizing shift in the voltage dependence of steady-state inactivation for Na_v_ 1.5. Interestingly, the C24A β3 mutant selectively attenuated the *V*_½_ for the inactivation shift by about 7 mV (10). This is comparable with the changes in inactivation gating shown by some Na_v_ channelopathy mutations (3). Our results thus suggest that the β3 subunits may influence the electrophysiological properties of Na_v_ channels by modulating their oligomeric status. Our model differs notably from the current consensus, in which Na_v_ channels are assumed to associate as single α and β subunits (4, 5). It does, however, provide a new framework both to re-interpret some aspects of Na_v_ channel behavior and to predict others.

**FIGURE 8.**
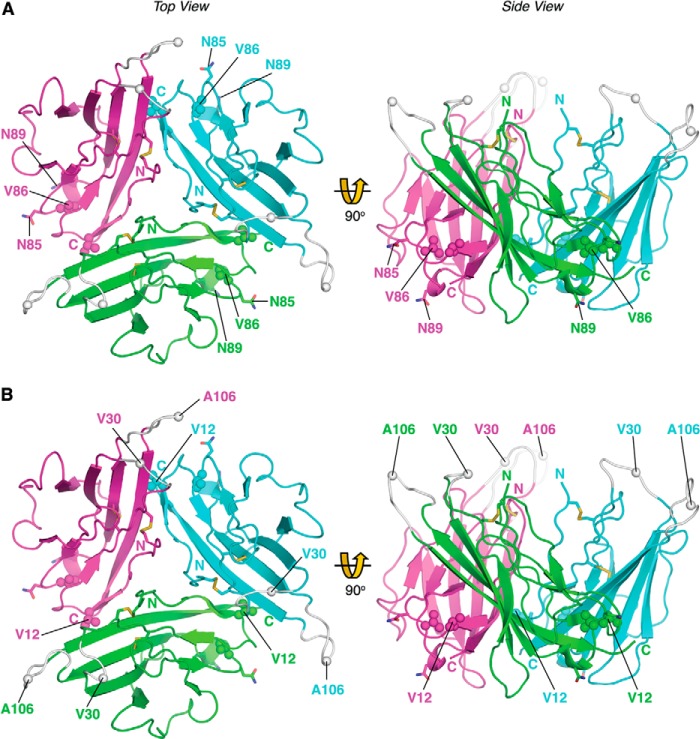
**Location of inherited β3 cardiopathy mutations.** The point mutations associated with cardiopathologies are shown as *spheres*: *A*, V86I. *B*, V12M, A106V, and V30G. Amino and carboxyl termini are labeled. *A*, asparagine residues of the *N*-linked glycosylation sites in close proximity to the Val-86 residue (Asn-85 and Asn-89) are shown as *sticks* and labeled. Note: as described under “Results,” the residues are numbered from the start of the mature protein, lacking the ER targeting signal. Loops that are not visible in the electron density maps due to local disorder are shown at their plausible theoretical positions in *gray*.

Several inherited cardiac arrhythmias are associated with point mutations in the human *SCN3B* gene (4). In four known cases, the mutations occur within the Ig domain (51–54). In the first example, residue Val-86 is changed to isoleucine (Fig. 8*A*) (54). Residue Val-86 is fully conserved between the β3 and β1 subunit isoforms and lies adjacent to potential *N*-linked glycosylation sites Asn-85 and Asn-89 (Figs. 1*A* and 8*A*). The Val-86 side chain provides tight internal hydrophobic packing. A more bulky isoleucine will perturb local contacts and could lead to misfolding. A second mutation changes residue Val-12 to methionine (Fig. 8*B*) (53). As noted above, residue Val-12 helps stabilize protomer association within the trimer; the larger methionine substitution is likely to perturb this interaction. The location of a pathological β3 mutation to a residue implicated in trimer stability provides further evidence for the functional importance of the trimer *in vivo*. In two further cases, residue Val-30 is mutated to glycine (51), and residue Ala-106 is mutated to valine (52). These mutations occur in adjacent loops that are unresolved in our structure due to local disorder. Nevertheless, their approximate positions can be inferred (Figs. 1*B* and 8*B*). Amino acid substitutions are well tolerated in Ig domain loops (40). In β3, the loops lie outside of, and point away from, the trimer interface. The mutations are thus unlikely to prevent trimer formation. Instead, they may identify one or more α subunit-binding sites. The A106V mutant (Fig. 8*B*) reduces peak current when co-expressed with Na_v_ 1.5 but does not affect surface expression of the α subunit. Interestingly, the mutant exerts a dominant negative effect on Na_v_ 1.5 gating when co-expressed with the wild-type β3 subunit (52). Dominant negative phenotypes often occur when mutant and wild-type subunits co-assemble to form an oligomer, with the mutant blocking the activity of every complex into which it is incorporated (55, 56). The dominant negative gating behavior of the A106V mutant could be explained if individual α subunits are functionally coupled within a β3-induced Na_v_ 1.5 trimer.

The β3 primary sequence is most closely related to that of β1 (4, 11). It is striking that most residues of the trimer interface, including the two critical cysteines, are fully conserved between β3 and β1 (Fig. 1*A*) (13). The β3 residue Arg-115 (Fig. 2*A*) is replaced by lysine in β1 (11), so a strong interaction could still form with Glu-4. The β1 subunit also contains a conserved arginine residue equivalent to Arg-100 (Fig. 2*A*, see above). In β1, separate mutations that change this arginine residue to leucine and cysteine are associated with inherited epilepsy syndromes (57, 58). However, residue Pro-6 of the β3 trimer interface is replaced with aspartic acid in β1 (11). Unless this charged residue can be neutralized by the formation of a salt bridge, the replacement of Pro-6 with Asp-6 would lead to electrostatic repulsion between β1 protomers if they adopted a trimer with exactly the same geometry as β3. We therefore predict that although the β1 subunit Ig domain may trimerize, the protomer organization will probably be different compared with β3. Mutations of acidic residues at the β1 amino terminus, including Glu-4, reduced the β1-dependent acceleration of Na_v_ 1.2 inactivation (59). In addition, the Cys-24-equivalent residue has been mutated in β1, leading to selective effects on Na_v_ channel inactivation and activation (60). We suggest that these β1 mutations may act by inhibiting α subunit oligomerization. Interestingly, it has recently been shown that the β1 subunit does indeed promote the co-assembly of multiple Na_v_ 1.5 α subunits in HEK293 cells (55).

The β2 and β4 subunit sequences are more closely related to each other than either is to β1 or β3 (4, 6). There is little sequence similarity between the β3 trimer interface and the equivalent regions of β2 or β4. An intramolecular disulfide bond corresponding to Cys-2–24 cannot form in either β2 or β4 because both lack the Cys-2-equivalent residue (38, 39). In β2, and probably also β4, the remaining cysteine forms an intersubunit disulfide bond with the α subunit (61). The atomic resolution structure of the β4 subunit Ig domain has recently been published (62). Although the overall Ig domain folds are similar to β3, there are also some very striking differences. In particular, and as expected from the sequence disparity noted above, the β4 subunit does not appear to form oligomers. Hence β4 (and probably β2) may not cross-link Na_v_ channel α subunits. This indicates a fundamental structural and possibly functional difference between the β1 and β3 subunits on the one hand and the β2 and β4 subunits on the other hand. Perhaps varying expression of the two structural classes of β subunits can differentially modulate Na_v_ channel clustering on the plasma membrane.

In conclusion, our results indicate a more complex picture of Na_v_ α/β subunit interaction than has been previously assumed, in which β3 subunits may play a role in facilitating Na_v_ channel oligomerization. This work provides new insights into the structure and function of the Na_v_ β3 subunit.

## Footnotes

9 The abbreviations used are:

ER: endoplasmic reticulum
CHES: 2-(cyclohexylamino)ethanesulfonic acid
MR: molecular replacement
AUC: analytical ultracentrifugation
AFM: atomic force microscopy
NCS: noncrystallographic symmetry
PALM: photoactivated localization microscopy
FPALM: fluorescence PALM
TIRF: total internal reflection fluorescence.
